# Echo time-dependent observed T1 and quantitative perfusion in chronic obstructive pulmonary disease using magnetic resonance imaging

**DOI:** 10.3389/fmed.2023.1254003

**Published:** 2024-01-05

**Authors:** Simon M. F. Triphan, Marilisa Konietzke, Jürgen Biederer, Monika Eichinger, Claus F. Vogelmeier, Rudolf A. Jörres, Hans-Ulrich Kauczor, Claus P. Heußel, Bertram J. Jobst, Mark O. Wielpütz, on behalf of the COSYCONET study group

**Affiliations:** ^1^Department of Diagnostic and Interventional Radiology, Subdivision of Pulmonary Imaging, University Hospital of Heidelberg, Heidelberg, Germany; ^2^Translational Lung Research Center Heidelberg (TLRC), Member of the German Center for Lung Research (DZL), Heidelberg, Germany; ^3^Boehringer Ingelheim Pharma GmbH and Co. KG, Biberach an der Riß, Germany; ^4^Faculty of Medicine, University of Latvia, Riga, Latvia; ^5^Faculty of Medicine, Christian-Albrechts-Universität zu Kiel, Kiel, Germany; ^6^Department of Diagnostic and Interventional Radiology With Nuclear Medicine, Thoraxklinik at the University Hospital of Heidelberg, Heidelberg, Germany; ^7^Department of Medicine, Pulmonary and Critical Care Medicine, Philipps-University of Marburg (UMR), Member of the German Center for Lung Research (DZL), Marburg, Germany; ^8^Institute and Outpatient Clinic for Occupational, Social and Environmental Medicine, Comprehensive Pneumology Center Munich (CPC-M), Member of the German Center for Lung Research (DZL), University Hospital, Ludwig Maximilians University (LMU) Munich, Munich, Germany

**Keywords:** magnetic resonance imaging, functional lung imaging, T1 mapping, lung T1, chronic obstructive pulmonary disease, dynamic contrast enhancement, quantitative perfusion

## Abstract

**Introduction:**

Due to hypoxic vasoconstriction, perfusion is interesting in the lungs. Magnetic Resonance Imaging (MRI) perfusion imaging based on Dynamic Contrast Enhancement (DCE) has been demonstrated in patients with Chronic Obstructive Pulmonary Diseases (COPD) using visual scores, and quantification methods were recently developed further. Inter-patient correlations of echo time-dependent observed T_1_ [T_1_(TE)] have been shown with perfusion scores, pulmonary function testing, and quantitative computed tomography. Here, we examined T_1_(TE) quantification and quantitative perfusion MRI together and investigated both inter-patient and local correlations between T_1_(TE) and quantitative perfusion.

**Methods:**

22 patients (age 68.0 ± 6.2) with COPD were examined using morphological MRI, inversion recovery multi-echo 2D ultra-short TE (UTE) in 1–2 slices for T_1_(TE) mapping, and 4D Time-resolved angiography With Stochastic Trajectories (TWIST) for DCE. T_1_(TE) maps were calculated from 2D UTE at five TEs from 70 to 2,300 μs. Pulmonary Blood Flow (PBF) and perfusion defect (QDP) maps were produced from DCE measurements. Lungs were automatically segmented on UTE images and morphological MRI and these segmentations registered to DCE images. DCE images were separately registered to UTE in corresponding slices and divided into corresponding subdivisions. Spearman’s correlation coefficients were calculated for inter-patient correlations using the entire segmented slices and for local correlations separately using registered images and subdivisions for each TE. Median T_1_(TE) in normal and defect areas according to QDP maps were compared.

**Results:**

Inter-patient correlations were strongest on average at TE_2_ = 500 μs, reaching up to |ρ| = 0.64 for T_1_ with PBF and |ρ| = 0.76 with QDP. Generally, local correlations of T_1_ with PBF were weaker at TE_2_ than at TE_1_ or TE_3_ and with maximum values of |ρ| = 0.66 (from registration) and |ρ| = 0.69 (from subdivision). In 18 patients, T_1_ was shorter in defect areas than in normal areas, with the relative difference smallest at TE_2_.

**Discussion:**

The inter-patient correlations of T_1_ with PBF and QDP found show similar strength and TE-dependence as those previously reported for visual perfusion scores and quantitative computed tomography. The local correlations and median T_1_ suggest that not only base T_1_ but also the TE-dependence of observed T_1_ in normal areas is closer to that found previously in healthy volunteers than in defect areas.

## 1 Introduction

Monitoring disease progression and therapy response in chronic obstructive pulmonary disease (COPD) is commonly based on pulmonary function testing (PFT), which provides global measures for lung function impairment (1). Computed tomography (CT) is used for the regional assessment of lung structure and distinction of COPD phenotypes (2). Magnetic resonance imaging (MRI) of the lungs is particularly challenging due to the low proton density in lung parenchyma, respiratory motion and especially the extremely short T_2_* relaxation time caused by the structure of the lungs (3). MRI was suggested as a radiation-free, non-invasive alternative to PFT and CT and has the additional benefit of techniques for the regional assessment of functional impairment. MRI methods based on image registration and the oscillation of signal intensity allow for the quantification of pulmonary perfusion and ventilation (4, 5). However, arguably the most practical and established approach for functional lung MRI in clinical routine is dynamic contrast enhanced (DCE) MRI. In the lungs, local perfusion is regulated by hypoxic pulmonary vasoconstriction which links perfusion directly to ventilation. Accordingly, the detection of perfusion defects can thus be used to identify abnormal ventilation as long as ventilation/perfusion mismatches can be disregarded. Reduction of the pulmonary vascular bed in emphysema also results in perfusion defects. A visual scoring system for DCE-MRI by radiologist readers has been demonstrated to provide a measure of functional lung abnormality that correlates well with other clinical metrics, including lung function tests in COPD and cystic fibrosis (6–10).

The determination of this visual score requires trained radiologists and is simultaneously limited in spatial resolution to the level of lung lobes. It is also severely limited in numerical resolution since scores cannot practically have fine graduations and it also essentially disregards the difference in volume between lung lobes. Quantification of perfusion from DCE-MRI data has been established in other organs and explored in the lungs as well (11, 12). For perfusion quantification, both the calculation of perfusion metrics like pulmonary blood flow (PBF), pulmonary blood volume (PBV) and mean transit time (MTT) as well as the determination of the extent of defects have been shown (13). While these have been examined mainly as global metrics for the entire lung, especially considering the fraction of defects as perfusion defects in percent (QDP), they are also available locally for each image voxel (14).

T_1_ quantification in the lungs was originally pursued in the context of oxygen-enhanced imaging (15). In these studies, significantly lower baseline lung T_1_ was found in patients with COPD even without oxygen supplementation, and median T_1_ was found to correlate with the visual MRI perfusion score (8). In subsequent work, it was further demonstrated that observed lung T_1_ [T_1_(TE)] is dependent on the echo time (TE) of the measurement. It was subsequently assumed that T_1_(TE) reflects different compartments of lung tissue protons inside each imaging voxel (protons in blood and extravascular protons), since the inter-patient correlations between T_1_(TE) and the MRI morphology and MRI perfusion score as well as other metrics vary with TE in COPD and cystic fibrosis (16–18). It was hypothesized that T_1_(TE) has the potential to differentiate tissue abnormalities such as inflammation or emphysema from perfusion abnormalities on the level of image voxels without the need for contrast material. Previous studies have so far examined the relationship between T_1_(TE) and DCE-MRI perfusion using only visual MRI perfusion scores on the level of the entire lungs for median T_1_, and on the level of lobes for visually scored T_1_ (8, 17, 18). The present work was conducted to further investigate the relationship of T_1_(TE) with quantitative perfusion metrics on a local level including voxel-wise correlations.

## 2 Methods

### 2.1 Patient characteristics

The study included participants of a single study center within the German multicenter COPD cohort study COSYCONET (“COPD and SYstemic consequences-COmorbidities NETwork,” ClinicalTrials.gov Identifier: NCT01245933) (19). The overarching COSYCONET study as well as the part of the imaging-based sub-study [“Image-based structural and functional phenotyping of the COSYCONET cohort using MRI and CT (MR-COPD),” ClinicalTrials.gov Identifier: NCT02629432] described here were approved by the responsible ethics committees of the coordinating centers [Institutional Review Board of the Medical Faculty of the University of University of Marburg (200/09) and of the University of Heidelberg (S-656/2012), Germany]. The participants of the present sub-study gave their written informed consent to undergo extensive clinical assessment including lung function testing, non-contrast CT, and morpho-functional MRI. The patient characteristics are summarized in Table 1. Some patients were included in separate previous reports, which did not assess the analyses made in the present study (13, 18).

**Table 1:** Patient characteristics.

Total no. of patients	22
Sex	12 female, 10 male
Age (years)	68.0 ± 6.2 (51.0–77.0)
Weight (kg)	77.3 ± 16.2 (49.0–115.0)
Height (cm)	169.8 ± 6.8 (156.0–181.0)
FEV1% predicted	59.9 ± 15.2 (34.0–84.0)
GOLD stage	1.6 ± 1.3 (0–4)

Values are given as mean ± standard deviation with the range in parentheses.

### 2.2 Magnetic resonance imaging acquisition

All measurements were performed on the same 1.5 T scanner (Magnetom Aera, Siemens Healthineers, Erlangen, Germany). A standardized protocol was employed including T_1_- and T_2_-weighted morphological MRI sequences. Of those, the T_1_-weighted morphological Volumetric Interpolated Breath-hold Examination (VIBE) measurements were used for segmentation. Imaging parameters for those were: Transverse VIBE: TR = 2.61 ms, TE = 1.69 ms, Field of View (FoV) 400 × 300 × 4 mm^3^, matrix size 320 × 240 × 88. Coronal VIBE: TR = 3.35 ms, TE = 1.63 ms, FoV 400 × 400 × 4 mm^3^, matrix size 288 × 288 × 56. A keyhole-based 3D gradient echo sequence [Time-resolved angiography With Stochastic Trajectories (TWIST), Siemens Healthineers, Erlangen, Germany], was used for DCE perfusion imaging, resulting in 1.7 s effective time resolution over 20 3D image timepoints. Imaging parameters were: up to 56 slices at 5 mm thickness, matrix size 208 × 256, FoV 366 × 450 mm^2^, TE 0.76 ms, and flip angle 20°. DCE measurements utilized intravenous contrast injection of 2 mL Gadobutrol 1 mmoL/mL (Gadovist, Bayer Vital, Leverkusen, Germany) injected at a rate of 4 mL/s, followed by a 30 mL 0.9% NaCl chaser. Both VIBE and TWIST were acquired in inspiratory breath-holds.

T_1_(TE) mapping was based on an inversion recovery multi-echo 2D ultra-short TE sequence, applied directly before contrast injection (20). Echos at TE_1-5_ = 70, 500, 1,200, 1,650, and 2,300 μs were acquired split into two separate measurements with TE_1_, TE_3_, and TE_5_ in the first and TE_2_ and TE_4_ in the second measurement. Imaging parameters were: One or two coronal slices at 15 mm thickness, matrix size 128 × 128, FoV 500 × 500 mm^2^, TR = 5 ms, flip angle 6°, and total acquisition time 4 min (1 slice) or 8 min (2 slices). To cover as much lung parenchyma as possible, the first slice was placed through the descending aorta and the second slice 30 mm ventral. For each measurement, a total of 6,000 radial spokes in a golden angle distribution were acquired, divided into blocks starting with an adiabatic inversion pulse followed by 300 radial spokes and separated by 3 s delays. These were then sorted by inversion time (TI), split using a sliding window 120 spokes wide with 60 spokes step-width and reconstructed using a non-uniform Fourier transform implemented in MATLAB (Mathworks, Natick, United States). To achieve ultra-short echo times, pairs of half sinc-pulses with opposing slice-selection gradients, 1,250 μs pulse duration and time-bandwidth-product 2 were employed. For each TE, T_1_ maps were calculated per voxel from the time-course along TI, based on the UTE images in each slice (21). UTE measurements were acquired during free breathing and gated to expiration using 50% of data, as described in Triphan et al. (20).

### 2.3 Magnetic resonance imaging analysis

Magnetic resonance images were segmented independently for UTE and DCE measurements: In UTE images, segmentation was performed on images at TE_3_ using simple region growing (for details, see the online supplement). Since the DCE images are three-dimensional and designed to maximize contrast between tissue with and without contrast agent, these were not segmented directly. Instead, a region growing-based segmentation algorithm was applied to morphological images with a higher resolution and the resulting segmentation masks were then registered to the DCE images, as described previously (13, 22).

For perfusion quantification, an arterial input function was detected automatically and then used to calculate a residual function R(t) by applying a voxel-wise deconvolution with the time-dependent signal in the DCE images (23). Pulmonary blood flow (PBF) maps were derived from R(t) by determining the maximum along the time-course in each individual voxel (24). To calculate defect classification maps and QDP, the following steps were performed: The timepoint of maximum contrast enhancement in the entire lungs (t_max_) was determined by taking the mean of R(t) in the entire lung for every timepoint. Otsu’s method was then applied to the values of R(t_max_) within the lungs to determine two thresholds (25). Defect classification maps were produced by classifying all voxels below the lower threshold as perfusion defects and the remainder as normal (13). The fraction of defect voxels within the segmented lungs on these maps relative to the total number of segmented voxels was defined as QDP. For all of the following steps, the three partitions of the 3D DCE images that correspond to the location of the UTE slice(s) were determined and averaged to produce a thicker projection image. Note that t_max_ and the classification thresholds were calculated based on the entire segmented lungs but QDP to be compared to T_1_(TE) only on these slices corresponding to UTE measurements.

**Figure 1:**
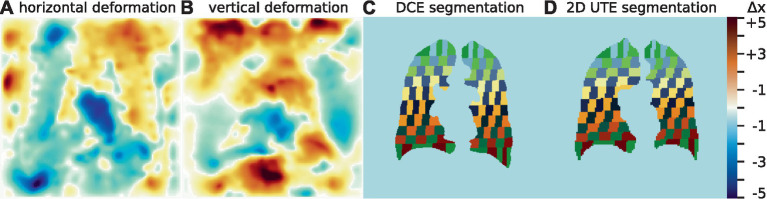
Example horizontal **(A)** and vertical **(B)** deformation field as determined by image registration, scaled in number of voxels. Segmentations with subdivided areas for a DCE image **(C)** and the corresponding 2D UTE image **(D)**. Areas that correspond to each other in the subdivision are drawn in identical colors.

To facilitate local (intra-patient) comparison of T_1_(TE) and quantitative perfusion maps, two approaches were investigated: (1) DCE images were averaged over their entire time-course and registered onto the corresponding UTE images using Advanced Normalization Tools (ANTs) (26) using the same parameters for all patients. The deformation fields produced by this registration were then applied to the PBF and defect classification maps as well as the lung segmentations derived from morphological imaging to allow for voxel-wise comparisons. An example of these deformation fields is given in Figures 1A,B. (2) On each slice, the left and right lungs were individually subdivided into areas with the following approach: Each lung was subdivided into 10 strips of equal volume vertically and then each of these strips was separated into 10 blocks of equal volume horizontally. By applying this subdivision algorithm on the segmentations of the DCE images and the UTE independently, this approach yields subdivided areas for both different resolutions and different respiratory states, which nevertheless correspond to the same physical volumes in the lungs and thus allow for correlations and comparisons without a registration step. An example of this subdivision is shown in Figures 1C,D.

### 2.4 Statistical analyses

Inter-patient correlations were calculated for median T_1_(TE) with median PBF (ρ_IPBF_) and QDP (ρ_IQDP_) within the segmented slices. For each patient, Spearman’s correlation coefficients between T_1_(TE) and PBF were calculated separately for registered voxels (ρ_regPBF_) and subdivided areas (ρ_subPBF_), respectively. Registered voxels in UTE were classified as normal perfusion or perfusion defect according to the corresponding classification map from DCE, and the median T_1_(TE) in each fraction was compared by Wilcoxon rank-sum tests. For subdivided areas, the fraction of defect voxels (i.e., local QDP) in each subdivided area was correlated with median T_1_(TE) in the corresponding area, analogous to PBF, as the local coefficient ρ_subQDP_. A value of *p* <0.05 was considered statistically significant.

## 3 Results

**Figure 2:**
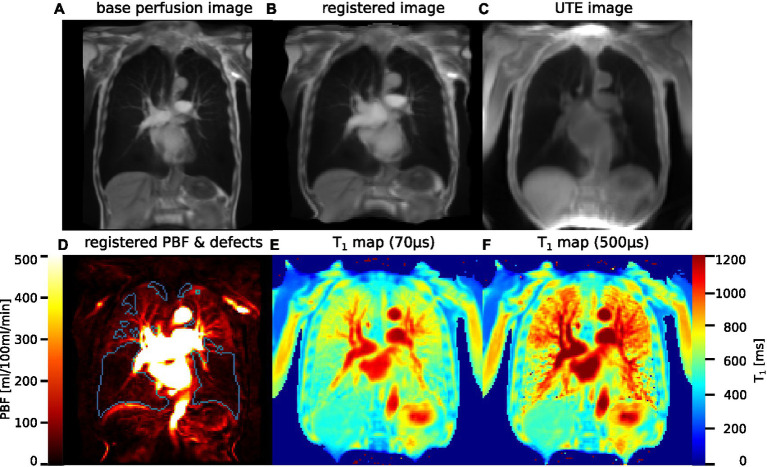
**(A)** Original image from DCE MRI, averaged over all timepoints and three slices. **(B)** The same image, registered to the UTE image. **(C)** Image derived from inversion recovery UTE that was used as registration target. **(D)** PBF map after application of the distortion field produced by the image registration, with perfusion defects delineated in blue. **(E)** T_1_ map acquired using UTE at TE_1_ = 70 μs. **(F)** T_1_ map at TE_2_ = 500 μs.

Suitable UTE and DCE measurements were available from 22 patients. In four patients, one of the two measurements comprising the UTE acquisition was mismatched in one slice or insufficient time was available to acquire both slices. Figure 2 shows an example set of base images and parameter maps in one patient.

**Figure 3:**
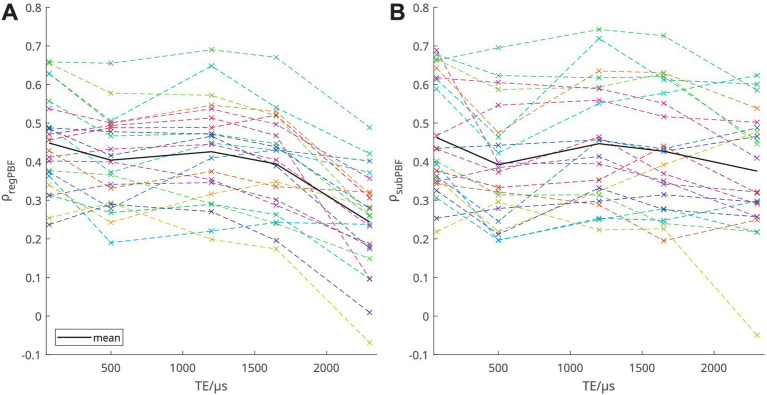
Spearman’s correlation coefficients for the correlation of T_1_(TE) with PBF, **(A)** voxel-wise correlation based on image registration and **(B)** based on subdivisions, respectively. Each patient is shown with the same color in both graphs.

The coefficients of the inter-patient correlation of T_1_(TE) with median PBF for all corresponding lung slices were ρ_IPBF1-5_ = 0.56, 0.64, 0.59, 0.60, and 0.29 at TE_1-5_ and the respective correlations of T_1_(TE) with QDP were ρ_IQDP1-5_ = −0.56, −0.76, −0.63, −0.53, and −0.26. Of these, only the correlations at TE_1-4_ were significant (at *p* < 0.02). Table 2 shows the mean local correlation coefficients of T_1_(TE) with PBF based on both methods for associating UTE with DCE images, as well as the correlation of T_1_(TE) with QDP. The values of ρ_regPBF_ and ρ_subPBF_ for individual patients are also shown in Figure 3 to illustrate the inter-patient variation.

**Table 2:** Mean local correlations.

TE	70 μs	500 μs	1,200 μs	1,650 μs	2,300 μs
ρ_regPBF_	0.44 ± 0.13 (22)	0.39 ± 0.12 (22)	0.42 ± 0.13 (22)	0.39 ± 0.13 (22)	0.24 ± 0.13 (21)
ρ_subPBF_	0.46 ± 0.15 (22)	0.39 ± 0.15 (22)	0.45 ± 0.16 (22)	0.43 ± 0.16 (22)	0.38 ± 0.16 (21)
ρ_subQDP_	−0.33 ± 0.16 (18)	−0.21 ± 0.16 (12)	−0.23 ± 0.17 (15)	−0.22 ± 0.15 (16)	−0.17 ± 0.16 (15)

Mean local correlations of T_1_(TE) with PBF, based on image registration for voxel-wise correlation (ρ_regPBF_) and based on subdivisions (ρ_subPBF_), as well as with QDP based on subdivisions (ρ_subQDP_). Values are given as means with standard deviations and the number of patients in whom the correlation was significant in brackets.

**Figure 4:**
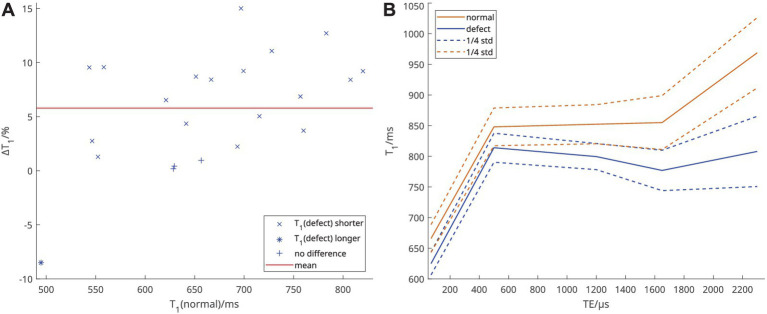
**(A)** Relative difference of T_1_ at TE_1_ = 70 μs between voxels classified as perfusion defect and voxels classified as normal. Each point represents one individual. **(B)** Mean of individual median T_1_ over all patients at each TE, according to defect classification.

At TE_1_ = 70 μs, in 18 out of 22 patients, T_1_ was shorter in voxels classified as perfusion defect when compared to the rest of the lung voxels classified as normal perfusion in the respective individual. In three patients, no significant difference was found and in one patient, T_1_ was longer in the defect area. While, as shown in Figure 4A, T_1_ in normal (non-defect) areas and ΔT_1_, the relative difference, varied strongly among patients, median T_1_ in defect areas was shorter at all TEs when considered over all patients. Notably, as shown in Figure 4B, mean ΔT_1_ was smaller at TE_2_ than at TE_1_ and TE_3_, with ΔT_1_ = 6.2, 4.0, 6.2, 9.1, and 16.6%, similar to the local correlations observed.

As demonstrated in Figure 2, visual inspection of parameter maps suggested that stronger local correlations appeared in patients where areas with clearly recognizable perfusion and longer T_1_ remained. To investigate this observation, the inter-patient correlation between median T_1_(TE_1_) and the local correlation coefficients of T_1_ and PBF was calculated. At TE_1_ = 70 μs, this was ρ_regcorr_ = 0.68 when using registration and ρ_subcorr_ = 0.32 when using subdivided areas.

## 4 Discussion

In this work, inter-patient correlations of T_1_(TE) with quantitative perfusion measures showed the strongest correlation coefficient at TE_2_ = 500 μs. Local voxel-wise correlations instead showed the strongest correlations at TE_1_ = 70 μs and in fact weaker correlations at TE_2_ than at TE_3_ = 1,200 μs. Finally, at TE_1_, in 18 out of 22 patients, T_1_ was shorter in voxels classified as defect than in normal areas.

The visual score for perfusion MRI has been established in COPD and cystic fibrosis, and were shown to correlate with clinical metrics (6, 10) and with T_1_ at conventional echo times (8). Subsequently, it was demonstrated that the inter-patient correlation of median T_1_(TE) in the entire lungs with this perfusion score and quantitative CT parameters depends on TE. Specifically, the strongest correlation was found at TE = 500 μs (18). This agrees with inter-patient correlations of T_1_(TE) with quantitative perfusion measures shown in this work which, also gave the strongest correlation at TE_2_.

Perfusion defects in percent was developed to compensate for the vulnerabilities in the quantification of PBF related to non-linearity of Gadolinium-concentration with MRI signal, artifacts, limited temporal resolution, and low SNR in the lung parenchyma (which is particularly low in diseased areas of the lung parenchyma) (14, 27, 28). This is supported by stronger inter-patient correlations between QDP and the visual perfusion score than were found with visual perfusion scores previously (13). In our present work, the inter-patient correlation between T_1_(TE) and QDP was also stronger at TE_2_ and stronger than with PBF. However, while the correlations of T_1_ with PBF based on subdivision were slightly stronger than those based on image registration, only very weak correlations of T_1_ with local QDP within subdivisions were found. This is most likely to due to many subdivided areas containing only defect or only normal voxels and thus not describing larger defect areas well. QDP derived from DCE using a different algorithm was found to locally match QDP derived from contrast-free PREFUL MRI (28). QDP derived from T_1_(TE) (rather than DCE-MRI) was shown to correlate similarly to T_1_(TE) itself in patients with *CF* (17), but not in patients in COPD (18). Thus, we did not re-investigate defect classification based on T_1_(TE) in the present work.

Unlike the inter-patient correlations, the local correlations found in the present study were highest at TE_1_ and higher at TE_3_ than at TE_2_. This can be attributed to lung areas more affected by disease having not only shorter T_1_ but also exhibiting a different dependence of T_1_ on TE. This is supported by the behavior of T_1_ in areas classified as perfusion defect or normal, when averaged over the patient collective: There, the average difference between these areas decreased from TE_1_ to TE_2_ and then increased again at TE_3_. This behavior becomes clearer when considering the data published previously comparing patients with COPD to healthy volunteers (18). There, the TE-dependence of T_1_ was much more gradual and observable at longer TEs in healthy volunteers, while the observed T_1_ in patients with COPD did not notably increase further after TE = 500 μs, which also meant that the separation between these groups was smallest at TE = 500 μs. Assuming that the TE-dependence of T_1_ in areas classified as normal through perfusion is closer to that in healthy volunteers and in defect areas closer to that in the patient group, this is consistent with the results seen in this work. It should however be noted that in the present collective areas classified as normal are still quite abnormal in T_1_ compared to values observed in healthy volunteers. Thus, our results show that both base T_1_ and its TE-dependence differ in areas of normal and defective perfusion. This further supports the hypothesis that it may be possible to separate the effects of local tissue changes and perfusion on T_1_ and investigate both with a single measurement. However, this is not yet possible with the current approach.

A large variation of local correlations was observed, with the strongest observed local correlations reaching the same magnitude as the inter-patient correlations. The overall visual impression of T_1_ and PBF parameter maps was that strong correlations were mainly present in patients with some volume of remaining functional lung tissue. In those cases, a notable spread of perfusion values exists that can be correlated in the first place. As such, we assumed that low local correlations might indicate relatively uniformly severely damaged lungs and thus also short T_1_. In order to confirm this assumption, we calculated the inter-patient correlation of median T_1_(TE_1_) with local correlation coefficients in each patient. That this correlation was significant supports the assertion that weak local correlations were primarily found in patients with more damaged lungs. However, this correlation was much weaker when based on subdivided areas. Since regions with long T_1_ are unlikely to coincide with the borders of subdivided areas, they can be assumed to describe these less well than the direct voxel-wise correlations. Similarly, in cases where a perfusion deficit was present all over the lung with corresponding short T_1_, these borders may matter less. At the same time, subdivided areas were less vulnerable to outlier values produced by quantification at very low SNR due to reduced proton density in severe emphysema. In further research, it may be worthwhile to investigate this in milder or presymptomatic cases of COPD, where a larger spread of values to correlate could be investigated. Additionally, MRI in general struggles in patients with severe emphysema simply due to lack of proton signal, though we might surmise that T_1_ mapping at TE_1_ = 70 μs should be better able to cope. However, in this work we were limited by the possibilities provided by DCE imaging.

Since in COPD patients, the TE-dependence of T_1_ is shifted to shorter TEs in comparison to healthy subjects, a tight spacing of TEs was necessary to depict the effects of interest in this work (16, 18): The TE-dependence of local and inter-patient correlations discussed here was visible at TE_1_ = 70 μs and TE_2_ = 500 μs, but it is not possible to measure T_1_ at both TEs in one TR due to the time required for the read dephasing gradient in between. Accordingly, the acquisition had to be split into two sets of echoes, which were implemented in separate measurements. This had the additional problem that the dc-signal used for respiratory gating is not necessarily aligned between those two measurements in case the patient changes how deep they breathe between them. Additionally, the two measurements could be misaligned due to operator error, making the entire slice unusable. After this study was concluded, we redesigned the sequence such that different sets of TEs could be acquired in alternating successive TRs which has neither of these disadvantages while still providing the required tight spacing of TEs.

The 4D MRI used for perfusion quantification was a single measurement, which meant that if the contrast injection was administered too early or late or the patient holding their breath for unsufficient time, it was entirely unusable for quantification. Since the UTE measurement was not part of the primary study protocol, we could only plan two slices and did not have time to retry in case of mistakes. However, these were separately acquired and each split into two measurements, as stated above. In cases where one slices was unusable due to operator error in at least one such sub-measurement, we decided to nevertheless use the remaining slice since the total number of patient measurements was so small. Thus, the UTE images being based on 2D slices was a major limitation of this work: a reliable 3D implementation of the multi-echo inversion recovery UTE would allow for a better quality of registration and give better coverage of the lungs, which would also provide improved comparability with perfusion images.

We investigated the approach to derive local correlations from subdivided areas in addition to image registration to avoid reliance on the registration algorithm, as it is not practically possible to guarantee a successful registration: The quality of image registration limits the comparability and thus voxel-wise correlation of parameter maps. The approach using subdivided areas was designed as an alternative that avoids this problem by increasing the size of spatial units to potentially correct for some imprecision in registration but only with the limitation that it is based on the assumption that both lungs have equal volume. This was implemented in order to be able to use the same number and pattern of subdivisions in each patient. In future work, the accuracy of local correlations could be increased by generating a specific subdivision pattern for each patient, possibly in 3D, which employs different number of subdivisions for each lung. Furthermore, the two segmentations may be subtly different beyond the issue of comparing two- with three-dimensional data. Finally, a relevant limitation for both approaches was that DCE images were acquired in inspiratory breath-holds, while UTE images were acquired during free breathing and reconstructed to expiration retrospectively.

As noted above, the approach discussed here relies entirely on using local perfusion as an indicator of lung function. However, even in early COPD stages, ventilation/perfusion (V/Q) mismatches have been reported, suggesting a blocked hypoxic pulmonary vasoconstriction mechanism to inhibit inflammation (29, 30). Nevertheless, there remains a significant association between ventilation and perfusion in COPD patients and correlation of the extent of V/Q mismatches with disease severity was relatively modest (31). To incorporate the effect of local V/Q mismatches, an additional direct measure of ventilation could be combined with perfusion imaging techniques. While non-contrast enhanced lung perfusion imaging methods have been introduced that also depict ventilation, it should be noted that the 2D UTE sequence used in this work was initially developed for oxygen-enhanced imaging since both T_1_ and T_2_* (which was not considered in this work but can be quantified from the same measurement) depend on the presence of molecular oxygen (dissolved in tissue and as gas in the alveoli, respectively) (4, 20). An additional acquisition while supplying subjects with oxygen could thus be used to determine local ventilation directly and thus investigate V/Q mismatches.

In conclusion, we have shown inter-patient correlations of T_1_(TE) with quantitative perfusion measures that are comparable to those described previously for T_1_(TE) with semi-quantitative visual perfusion scores from perfusion MRI and quantitative CT parameters. Local (intra-patient) correlations were much weaker on average, but the strongest correlations found reached a similar scale as the inter-patient correlation coefficients. Importantly, the TE-dependency of the local correlation coefficients implies that not only base T_1_ but also its TE-dependency in normal areas is closer to the behavior previously found in healthy volunteers than in defect areas. That the correlation coefficients themselves correlate with overall average T_1_ implies that weak local correlations would be observed in patients with few remaining areas of well-functioning lung tissue and thus low contrasts between local observations. Accordingly, it may be interesting to investigate DCE perfusion and T_1_(TE) in a patient cohort with less severe, earlier disease, both individually and in combination.

## Data availability statement

The original contributions presented in the study are included in the article/Supplementary material, further inquiries can be directed to the corresponding author.

## Ethics statement

The studies involving humans were approved by Institutional Review Board of the Medical Faculty of the University of Marburg (200/09) and of the University of Heidelberg (S-656/2012). The studies were conducted in accordance with the local legislation and institutional requirements. The participants provided their written informed consent to participate in this study.

## Author contributions

ST: Conceptualization, Data curation, Formal analysis, Investigation, Methodology, Software, Validation, Visualization, Writing – original draft, Writing – review & editing. MK: Conceptualization, Investigation, Methodology, Software, Writing – original draft, Writing – review & editing. JB: Conceptualization, Funding acquisition, Investigation, Methodology, Resources, Supervision, Writing – review & editing. ME: Conceptualization, Supervision, Writing – review & editing. CV: Conceptualization, Funding acquisition, Project administration, Resources, Writing – review & editing. RJ: Conceptualization, Funding acquisition, Project administration, Resources, Writing – review & editing. H-UK: Conceptualization, Funding acquisition, Investigation, Methodology, Project administration, Resources, Supervision, Writing – review & editing. CH: Conceptualization, Methodology, Project administration, Writing – review & editing. BJ: Conceptualization, Investigation, Methodology, Writing – review & editing. MW: Conceptualization, Investigation, Methodology, Project administration, Supervision, Writing – original draft, Writing – review & editing.

## Supplementary material

SUPPLEMENTARY FIGURE 1 Relative difference of T1 at TE1 to TE5 between voxels classified as perfusion defect and voxelsclassified as normal. Each point represents one individual. This corresponds to Figure 4A at all TE.

## References

[1] Hashimoto N Wakahara K Sakamoto K. The importance of appropriate diagnosis in the practical Management of Chronic Obstructive Pulmonary Disease. Diagnostics. (2021) 11. doi: 10.3390/diagnostics11040618, PMID: 33808229 PMC8067197

[2] Lynch DA Austin JHM Hogg JC Grenier PA Kauczor H-U Bankier AA. CT-definable subtypes of chronic obstructive pulmonary disease: a statement of the Fleischner society. Radiology. (2015) 277:192–205. doi: 10.1148/radiol.2015141579 25961632 PMC4613878

[3] Hatabu H Alsop DC Listerud J Bonnet M Gefter WB. T2* and proton density measurement of normal human lung parenchyma using submillisecond echo time gradient echo magnetic resonance imaging. Eur J Radiol. (1999) 29:245–52. doi: 10.1016/S0720-048X(98)00169-7, PMID: 10399610

[4] Bauman G Puderbach M Deimling M Jellus V Chefd'hotel C Dinkel J. Non-contrast-enhanced perfusion and ventilation assessment of the human lung by means of fourier decomposition in proton MRI. Magn Reson Med. (2009) 62:656–64. doi: 10.1002/mrm.22031, PMID: 19585597

[5] Bauman G Bieri O. Matrix pencil decomposition of time-resolved proton MRI for robust and improved assessment of pulmonary ventilation and perfusion. Magn Reson Med. (2017) 77:336–42. doi: 10.1002/mrm.26096, PMID: 26757102

[6] Eichinger M Optazaite D-E Kopp-Schneider A Hintze C Biederer J Niemann A. Morphologic and functional scoring of cystic fibrosis lung disease using MRI. Eur J Radiol. (2012) 81:1321–9. doi: 10.1016/j.ejrad.2011.02.045, PMID: 21429685

[7] Wielpütz MO Puderbach M Kopp-Schneider A Stahl M Fritzsching E Sommerburg O. Magnetic resonance imaging detects changes in structure and perfusion, and response to therapy in early cystic fibrosis lung disease. Am J Respir Crit Care Med. (2014) 189:956–65. doi: 10.1164/rccm.201309-1659OC, PMID: 24564281

[8] Jobst BJ Triphan SMF Sedlaczek O Anjorin A Kauczor HU Biederer J. Functional lung MRI in chronic obstructive pulmonary disease: comparison of T1 mapping, oxygen-enhanced T1 mapping and dynamic contrast enhanced perfusion. PLoS One. (2015) 10:e0121520. doi: 10.1371/journal.pone.0121520, PMID: 25822195 PMC4379151

[9] Stahl M Wielpütz MO Graeber SY Joachim MC Sommerburg O Kauczor H-U. Comparison of lung clearance index and magnetic resonance imaging for assessment of lung disease in children with cystic fibrosis. Am J Respir Crit Care Med. (2017) 195:349–59. doi: 10.1164/rccm.201604-0893OC, PMID: 27575911

[10] Wielpütz MO Eichinger M Wege S Eberhardt R Mall MA Kauczor H-U. Mid-term reproducibility of chest MRI in adults with clinically stable cystic fibrosis and chronic obstructive pulmonary disease. Am J Respir Crit Care Med. (2019) 200:103–7. doi: 10.1164/rccm.201812-2356LE, PMID: 30875236

[11] Hatabu H Tadamura E Levin DL Chen Q Li W Kim D. Quantitative assessment of pulmonary perfusion with dynamic contrast-enhanced MRI. Magnet Reson Med. (1999) 42:1033–8. doi: 10.1002/(sici)1522-2594(199912)42:6<1033:aid-mrm7>3.0.co;2-7 10571924

[12] Ohno Y Hatabu H Murase K Higashino T Kawamitsu H Watanabe H. Quantitative assessment of regional pulmonary perfusion in the entire lung using three-dimensional ultrafast dynamic contrast-enhanced magnetic resonance imaging: preliminary experience in 40 subjects. J Magn Reson Imaging. (2004) 20:353–65. doi: 10.1002/jmri.20137, PMID: 15332240

[13] Schiwek M Triphan SMF Biederer J Weinheimer O Eichinger M Vogelmeier CF. Quantification of pulmonary perfusion abnormalities using DCE-MRI in COPD: comparison with quantitative CT and pulmonary function. Eur Radiol. (2022) 32:1879–90. doi: 10.1007/s00330-021-08229-6, PMID: 34553255 PMC8831348

[14] Konietzke M Triphan SMF Eichinger M Bossert S Heller H Wege S. Unsupervised clustering algorithms improve the reproducibility of dynamic contrast-enhanced magnetic resonance imaging pulmonary perfusion quantification in muco-obstructive lung diseases. Front Med. (2022) 9:1022981. doi: 10.3389/fmed.2022.1022981, PMID: 36353218 PMC9637664

[15] Jakob PM Wang T Schultz G Hebestreit H Hebestreit A Hahn D. Assessment of human pulmonary function using oxygen-enhanced T1 imaging in patients with cystic fibrosis. Magn Reson Med. (2004) 51:1009–16. doi: 10.1002/mrm.20051 15122684

[16] Triphan SMF Jobst BJ Breuer FA Wielpütz MO Kauczor H-U Biederer J. Echo time dependence of observed T1 in the human lung. J Magn Reson Imaging. (2015) 42:610–6. doi: 10.1002/jmri.24840 25604043

[17] Triphan SMF Stahl M Jobst BJ Sommerburg O Kauczor H-U Schenk J-P. Echo time-dependence of observed lung T1 in patients with cystic fibrosis and correlation with clinical metrics. J Magn Reson Imaging. (2020) 52:1645–54. doi: 10.1002/jmri.27271, PMID: 32613717

[18] Triphan SMF Weinheimer O Gutberlet M Heußel CP Vogel-Claussen J Herth F. Echo time-dependent observed lung T1 in patients with chronic obstructive pulmonary disease in correlation with quantitative imaging and clinical indices. J Magn Reson Imaging. (2021) 54:1562–71. doi: 10.1002/jmri.27746, PMID: 34050576

[19] Karch A Vogelmeier C Welte T Bals R Kauczor H-U Biederer J. The German COPD cohort COSYCONET: aims, methods and descriptive analysis of the study population at baseline. Respir Med. (2016) 114:27–37. doi: 10.1016/j.rmed.2016.03.008, PMID: 27109808

[20] Triphan SMF Breuer FA Gensler D Kauczor H-U Jakob PM. Oxygen enhanced lung MRI by simultaneous measurement of T1 and T2* during free breathing using ultrashort TE. J Magn Reson Imaging. (2015) 41:1708–14. doi: 10.1002/jmri.24692, PMID: 25044618

[21] Deichmann R Haase A. Quantification of T1 values by SNAPSHOT-FLASH NMR imaging. J Magn Reson Imaging. (1992) 96:608–12. doi: 10.1016/0022-2364(92)90347-A

[22] Kohlmann P Strehlow J Jobst B Krass S Kuhnigk J-M Anjorin A. Automatic lung segmentation method for MRI-based lung perfusion studies of patients with chronic obstructive pulmonary disease. Int J Comput Assist Radiol Surg. (2015) 10:403–17. doi: 10.1007/s11548-014-1090-0, PMID: 24989967

[23] Kohlmann P Laue H Krass S Peitgen H-O (2011). “Fully-automatic determination of the arterial input function for dynamic contrast-enhanced pulmonary MR imaging” in *MIUA*.

[24] Sourbron S Dujardin M Makkat S Luypaert R. Pixel-by-pixel deconvolution of bolus-tracking data: optimization and implementation. Phys Med Biol. (2007) 52:429–47. doi: 10.1088/0031-9155/52/2/009, PMID: 17202625

[25] Otsu N. A threshold selection method from gray-level histograms. IEEE Trans Syst Man Cybern. (1979) 9:62–6. doi: 10.1109/TSMC.1979.4310076

[26] Avants BB Tustison N Song G. Advanced normalization tools (ANTS). Insight J. (2009) 2:1–35. doi: 10.54294/uvnhin

[27] Woodhouse N Wild JM Paley MNJ Fichele S Said Z Swift AJ. Combined helium-3/proton magnetic resonance imaging measurement of ventilated lung volumes in smokers compared to never-smokers. J Magn Reson Imaging. (2005) 21:365–9. doi: 10.1002/jmri.20290, PMID: 15779032

[28] Kaireit TF Voskrebenzev A Gutberlet M Freise J Jobst B Kauczor H-U. Comparison of quantitative regional perfusion-weighted phase resolved functional lung (PREFUL) MRI with dynamic gadolinium-enhanced regional pulmonary perfusion MRI in COPD patients. J Magn Reson Imaging. (2019) 49:1122–32. doi: 10.1002/jmri.26342, PMID: 30350440

[29] Agustí AG Barberá JA Roca J Wagner PD Guitart R Rodriguez-Roisín R. Hypoxic pulmonary vasoconstriction and gas exchange during exercise in chronic obstructive pulmonary disease. Chest. (1990) 97:268–75. doi: 10.1378/chest.97.2.268, PMID: 2298050

[30] Hoffman EA Chon D. Computed tomography studies of lung ventilation and perfusion. Proc Am Thorac Soc. (2005) 2:492–506. doi: 10.1513/pats.200509-099DS, PMID: 16352755 PMC2713338

[31] Rodríguez-Roisin R Drakulovic M Rodríguez DA Roca J Barberà JA Wagner PD. Ventilation-perfusion imbalance and chronic obstructive pulmonary disease staging severity. J Appl Physiol. (2009) 106:1902–8. doi: 10.1152/japplphysiol.00085.2009, PMID: 19372303

