# Supplementary figures and images for: Echo time-dependent observed T1 and quantitative perfusion in chronic obstructive pulmonary disease using magnetic resonance imaging
Source: Front Med (Lausanne). 2024 Jan 5;10:1254003. doi: 10.3389/fmed.2023.1254003 (PMC10797117; doi:10.3389/fmed.2023.1254003)

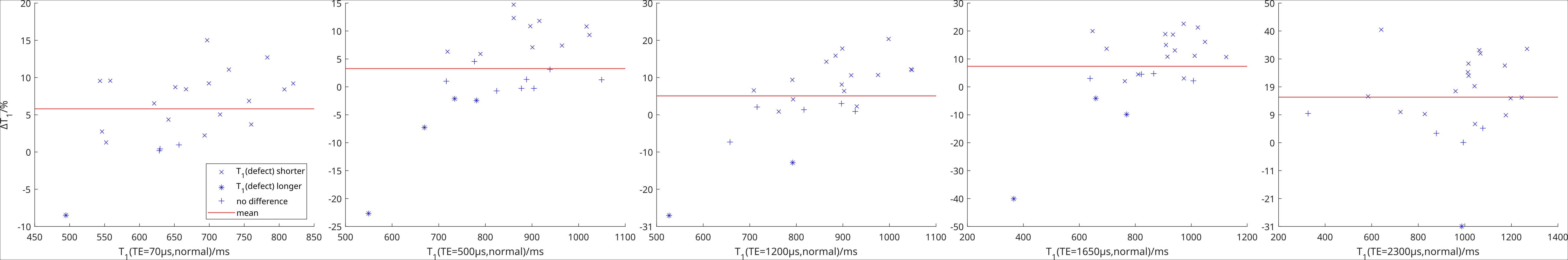

Supplement: SUPPLEMENTARY FIGURE 1 — Relative difference of T1 at TE1 to TE5 between voxels classified as perfusion defect and voxelsclassified as normal. Each point represents one individual. This corresponds to Figure 4A at all TE. [file Image_1.PNG]
