# Supplementary material for: Echo time-dependent observed T1 and quantitative perfusion in chronic obstructive pulmonary disease using magnetic resonance imaging
Source: Front Med (Lausanne). 2024 Jan 5;10:1254003. doi: 10.3389/fmed.2023.1254003 (PMC10797117; doi:10.3389/fmed.2023.1254003)
Supplement: Supplementary file 2 [file Table_1.pdf]

No	T_1/ms		p	ρ T_1 vs		
	defect	normal		PBF (reg)	PBF (sub)	QDP (sub)
1	678	693	0.005	0.43	0.34	-0.09
2	732	760	<0.001	0.63	0.64	-0.37
3	595	651	<0.001	0.34	0.36	-0.39
4	650	657	0.454+	0.25	0.22	-0.18
5	744	818	<0.001	0.64	0.67	-0.61
6	531	546	<0.001	0.38	0.40	-0.17
7	740	808	<0.001	0.66	0.66	-0.43
8	592	697	<0.001	0.56	0.68	-0.53
9	627	629	0.556+	0.31	0.31	-0.07
10	627	738	<0.001	0.47	0.61	-0.51
11	631	692	<0.001	0.43	0.59	-0.51
12	492	543	<0.001	0.36	0.39	-0.39
13	635	699	<0.001	0.37	0.36	-0.42
14	611	667	<0.001	0.49	0.43	-0.27
15	705	757	<0.001	0.48	0.33	-0.27
16	627	628	0.950+	0.24	0.25	-0.08
17	505	558	<0.001	0.31	0.35	-0.44
18	537	462	<0.001*	0.32	0.69	-0.36
19	647	728	<0.001	0.54	0.62	-0.23
20	545	552	0.048	0.41	0.47	-0.22
21	674	718	<0.001	0.45	0.43	-0.44
22	614	641	<0.001	0.46	0.38	-0.19

Table S1: Median T_1(TE_1=70µs) values in defect and normal areas as classified using perfusion MR quantification and Spearman's correlation coefficients of T_1 with PBF based on registered perfusion images, subdivided areas as well as for the correlation of T_1 with QDP. + indicates that T_1 in defect areas was not significantly different from the normal areas, * that it was longer.
